# Primary CNS Lymphoma in India: A 17-Year Experience From the All India Institute of Medical Sciences

**DOI:** 10.1200/JGO.18.00124

**Published:** 2019-02-27

**Authors:** Mukesh Patekar, Narayan Adhikari, Ahitagni Biswas, Vinod Raina, Lalit Kumar, Bidhu Kalyan Mohanti, Ajay Gogia, Atul Sharma, Atul Batra, Sameer Bakhshi, Ajay Garg, Sanjay Thulkar, Meher Chand Sharma, Sreenivas Vishnubhatla, Saphalta Baghmar, Ranjit Kumar Sahoo

**Affiliations:** ^1^ Dr B.R.A. Institute Rotary Cancer Hospital, New Delhi, India; ^2^ All India Institute of Medical Sciences, New Delhi, India

## Abstract

**PURPOSE:**

The information about the outcome of primary CNS lymphoma (PCNSL) in India is scarce, because there is no population-based or large hospital-based data.

**MATERIALS AND METHODS:**

This is a retrospective study that spanned 17 years (2001 to 2017) to study the outcome of PCNSL at the All India Institute of Medical Sciences (AIIMS), which is a tertiary care center in Northern India.

**RESULTS:**

Only one of 99 patients was positive for HIV serology. Diffuse large B-cell lymphoma was the most common histology (97.7%). The median patient age was 50 years (range, 13 to 70 years), and the ratio of men to women was 1.9. The median duration of symptoms before diagnosis was 3.5 months (range, 0.5 to 48 months), and 58.5% had a performance status (PS) of 3 or more. Multiple intracranial lesions were present in 81.8% of patients. Surgical resection was performed in 45%, and approximately 22% of patients were ineligible for treatment. Most patients (n = 73) were treated with high-dose methotrexate (HDMTX)–based regimens (ie, methotrexate, vincristine, and procarbazine with or without rituximab). Pharmacokinetic monitoring of methotrexate was not available at our center. HDMTX-related mortality was 3.9%. The median follow-up duration, event-free survival (EFS), and overall survival (OS) were 34 months, 20.4 months, and 31.7 months, respectively. Addition of rituximab (n = 27) to MVP resulted in a higher objective response rate (88.9% *v* 73.9% without rituximab; *P* = .12), complete remission (81.5% *v* 56.5%; *P* = .03), 2-year EFS (57.3% *v* 40.4%; *P* = .02), and 2-year OS (61.6% *v* 53.4%; *P* = .056).

**CONCLUSION:**

This is the largest study of PCNSL from India. The patients were immunocompetent and young but presented with a high-burden disease that precluded treatment in approximately 22%. The treatment with HDMTX appears safe without pharmacokinetic monitoring. The outcome is comparable to those observed in the West, and rituximab use showed additional benefit. There are notable barriers with respect to management of PCNSL in the real world, and efforts are required to improve the outcome more.

## INTRODUCTION

Primary CNS lymphoma (PCNSL) is a form of extranodal non-Hodgkin lymphoma (NHL) that is confined to brain, eyes, and—rarely—spinal cord or leptomeninges. More than 90% of these cases are classified as diffuse large B-cell lymphoma (DLBCL). It is a rare neoplasm that accounts for a fraction of NHL (approximately 1%) and all primary brain tumors (approximately 4%) in the United States and occurs predominantly in men of ages 60s or 70s, but it occurs one or two decades earlier in immunocompromised patients (eg, those with HIV).^1^ The prognosis in untreated patients is extremely poor (3 to 4 months) and modestly improved with whole-brain radiation therapy (WBRT; approximately 12 months); it is substantially improved (32 to 37 months) with high-dose methotrexate (HDMTX)–based chemotherapy and is improved even more by the addition of rituximab.^2-6^ However, this improvement in survival is not mirrored in patients treated outside of a clinical trial or in the population-based cohort studies; the discrepancy is a matter of concern.^7-9^

The management of PCNSL poses a unique challenge in resource-limited settings. First, because of its orphan-disease nature, the lymphoma could easily be missed despite its characteristic radiologic appearance.^10^ Second, unlike other brain neoplasms, surgical removal of the tumor is unnecessary and histopathologic diagnosis is mandatory by stereotactic needle biopsy, which is not routinely available at many centers.^10^ Third, PCNSL presents as a periventricular tumor, often with notable vasogenic edema and surrounding mass effect, and the use of corticosteroids to reduce cerebral edema is a common practice in patients with newly diagnosed brain tumors. Corticosteroids cause profound apoptosis of the lymphomatous cells and may obscure as well as delay the ability to make a definitive diagnosis^10,11^; corticosteroid use has the potential to affect the outcome by not only delaying the diagnosis but also by selecting out the drug-resistant clones. Finally, treatment with HDMTX is risky and requires intensive monitoring and experience.^12^

**CONTEXT**

**Key objective**
Despite numerous advances in the field of primary CNS lymphoma, there remain notable barriers in the management of this rare and aggressive brain neoplasm.
There are no population-based data for PCNSL in India. In such a scenario, large hospital-based data may be revealing.
This is a retrospective study that spanned 17 years, from 2001 to 2017, and was conducted at the All India Institute of Medical Sciences (AIIMS), which is a tertiary care center in the Northern India.
**Knowledge generated**
Patients were young and immunocompetent and presented with a high-burden disease at baseline.
High-dose methotrexate–based polychemotherapy is effective and can be safely given without pharmacokinetic monitoring.
The outcome of patients who could be treated appears comparable to that of patients from the West.
**Relevance**
This is the largest study of PCNSL from India representative of the experiences across all parts of the country and discusses the challenges faced in this country.

In India, PCNSL represents approximately 1% of all primary CNS neoplasms according to the hospital-based data reported by many centers, including ours.^13-16^ In this study, we attempted to characterize the clinical and pathologic profile and the outcome of patients with PCNSL who were treated during a period of 17 years at the All India Institute of Medical Sciences (AIIMS), which is a tertiary care center in Northern India. We also elaborate on the challenges faced during the management of the disease.

## MATERIALS AND METHODS

We examined the medical records of all patients diagnosed with PCNSL at our center between 2001 and 2017. Ethical clearance was obtained from the institutional ethical review committee. The date of biopsy was taken as the date of diagnosis. Contrast-enhanced magnetic resonance imaging was performed at diagnosis, after induction, after consolidation, then every 3 months during first 2 years, every 6 months for the next 3 years, and annually thereafter.

Each cycle of HDMTX was administered after admission as an inpatient. Each patient received intravenous hydration at a rate of 3 L/m^2^ and intravenous sodium bicarbonate for urine alkalization. After a urine pH of more than 7.5 had been maintained for two consecutive measures, HDMTX was started and infused over 3 hours. The hydration and alkalinization were maintained for the subsequent 48 to 72 hours. Leucovorin rescue was initiated 24 hours after methotrexate administration at a dosage of 20 mg intravenously (preferably) or orally every 6 hours for 12 doses. Serum creatinine, blood urea, and CBC were monitored daily. Pharmacokinetic monitoring of methotrexate could not be performed, because it is not routinely done at our center.

The standard dose of WBRT was 45 Gy in 25 fractions over 5 weeks (n = 50) by bilateral parallel opposed skull fields using a German Helmet portal with cobalt-60 gamma rays; the procedure is described in detail elsewhere.^17^ In a few patients (n = 10), reduced-dose WBRT (23.4 Gy in 13 fractions over 2.5 weeks) was administered, because these patient experienced complete response (CR) as part of a study protocol.^4,17,18^

χ^2^ or Fisher’s exact test was used to detect an association between categoric variables. Survival was estimated by the Kaplan-Meier method and compared using the log-rank test. Data were censored on February 15, 2018. A univariable Cox proportional hazard model followed by a multivariable Cox regression analysis were performed to identify the predictors of outcome. Event-free survival (EFS) was calculated from the date of diagnosis to date of disease relapse or progression or of death as a result of any cause. Overall survival (OS) was calculated from the date of diagnosis to date of death as a result of any cause. STATA/SE 13.0 (Stata Corp, College Station, TX) was used for statistical analysis.

## RESULTS

### Patient Characteristics

We identified 99 occurrences of PCNSL during the years 2001 to 2017 (Table 1). Only a single patient was positive for HIV serology. The median age at presentation was 50 years (range, 13 to 70 years), and the ratio of men to women was 1.9:1. The median duration of symptoms before diagnosis was 3.5 months (range, 0.5 to 48 months); common symptoms were motor abnormality (60.6%) followed by features of raised intracranial tension (49.5%) and abnormality of higher mental function (47.4%). Ocular and leptomeningeal involvement (positive CSF cytology) at presentation were found in 19.4% and 15.8% of patients, respectively. An Eastern Cooperative Oncology Group performance status (PS) of 3 or more at presentation was found in 58.5% of patients.

**TABLE 1:**
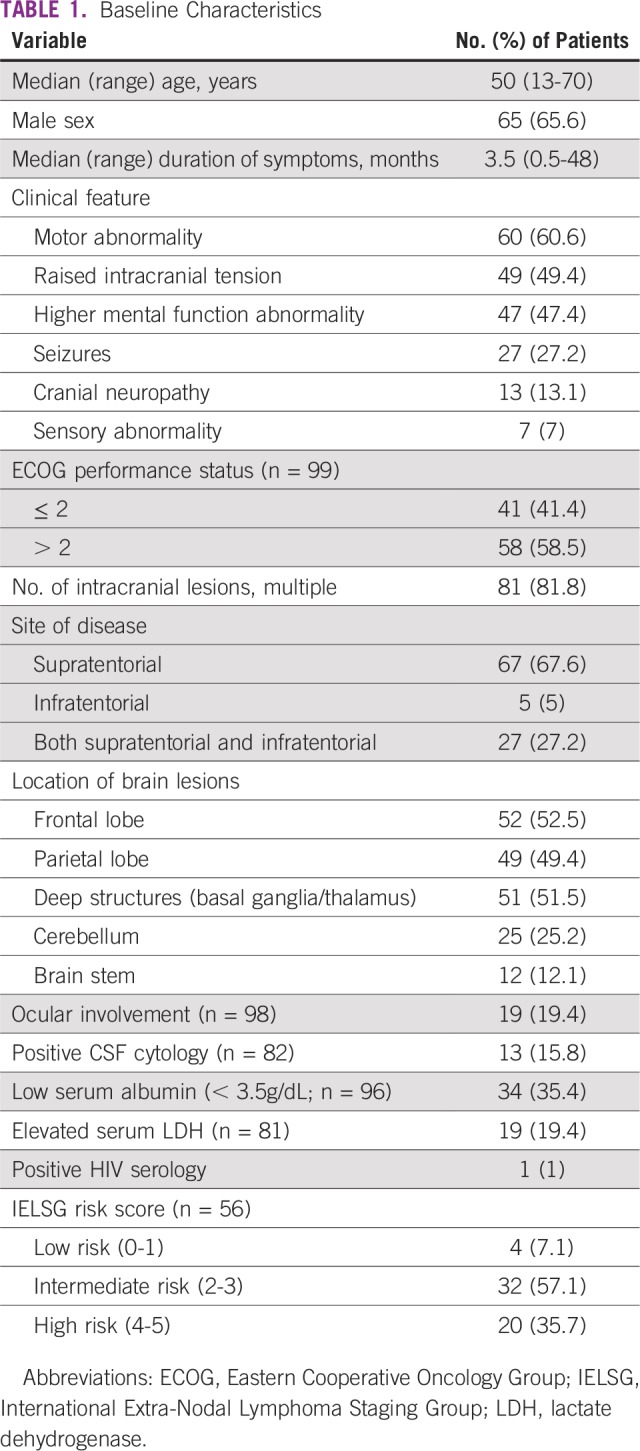
Baseline Characteristics

### Tumor Characteristics

The histopathology was DLBCL in 97.7% of patients (87 of 89 patients; Table 2). The histologic diagnosis was established by biopsy (stereotactic or open) in 49 patients and by surgery in 40 patients. In 10 patients (approximately 10%), the diagnosis was entirely based on the typical radiographic appearance, because biopsy could not be obtained or was inconclusive. Multiple intracranial lesions were found in 81.8% of patients; these occurred predominantly in the frontal lobe (52.53%) followed by the parietal lobe (49.49%), and involvement of deep structures (basal ganglia/thalamus) was seen in 51.5% of patients.

**TABLE 2:**
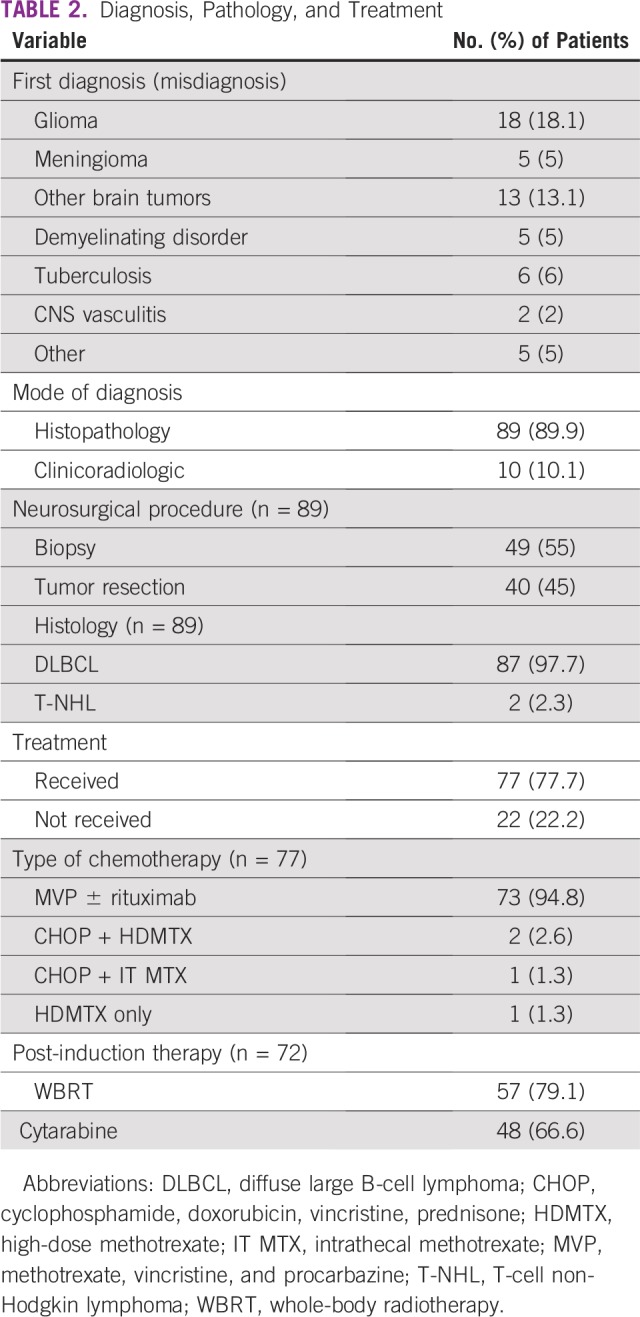
Diagnosis, Pathology, and Treatment

### Treatment and Outcome

**FIG 1:**
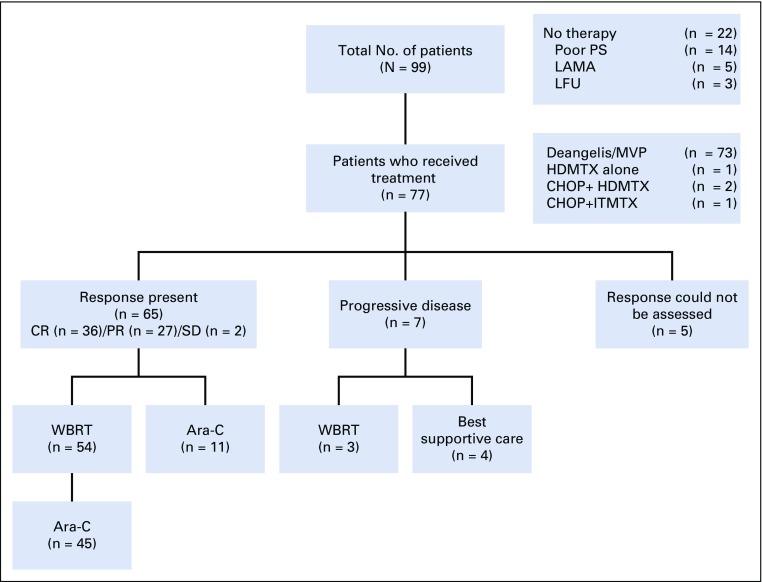
Flow diagram. Ara-C, cytarabine; CHOP, cyclophosphamide, doxorubicin, vincristine, prednisone, CR, complete response; HDMTX, high-dose methotrexate; IT MTX, intrathecal methotrexate; LAMA, left against medical advice; LFU, lost to follow-up; MVP, methotrexate, vincristine, and procarbazine; PR, partial response; SD, stable disease; WBRT, whole-body radiotherapy.

Twenty-two of 99 patients did not receive any therapy, so they were excluded from the outcome analysis (Fig 1). Of 77 patients, 73 patients received MVP ± R (HDMTX, vincristine, and procarbazine, with or without rituximab), as suggested by DeAngelis et al,^3^ or its modification (Table 2).^3,4^ The median numbers of MVP ± R cycles was five (range, two to five cycles). Rituximab was used in patients who could afford it (n = 27).

The objective response rate (ORR) to chemotherapy was 81.8%; 46.8% had CR. Seven (9%) of 77 patients had progressive disease (PD); among them, three could receive WBRT as salvage. The final response after consolidation (WBRT and/or high-dose cytarabine) was 80.5%, and 67.5% had CR. Nine patients had PD, and all except one patient died as a result. In five patients, WBRT was avoided in view of advanced age (older than 60 years). In a single case, the reason for the omission of WBRT could not be ascertained.

**FIG 2:**
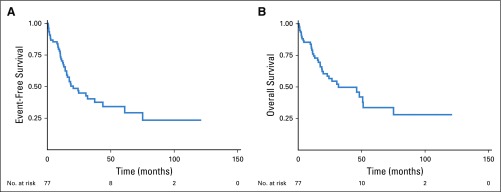
Kaplan-Meier survival estimates: (A) event-free survival; and (B) overall survival.

The median duration of follow-up for the whole cohort was 34 months. The median EFS and OS were 20.4 months and 31.7 months, respectively (Figs 2A and 2B). In the multivariable analysis, the absence of resection, infratentorial involvement, and low serum albumin (< 3.5 g/dL) were independent predictors of both EFS and OS (Tables 3 and 4).

**TABLE 3:**
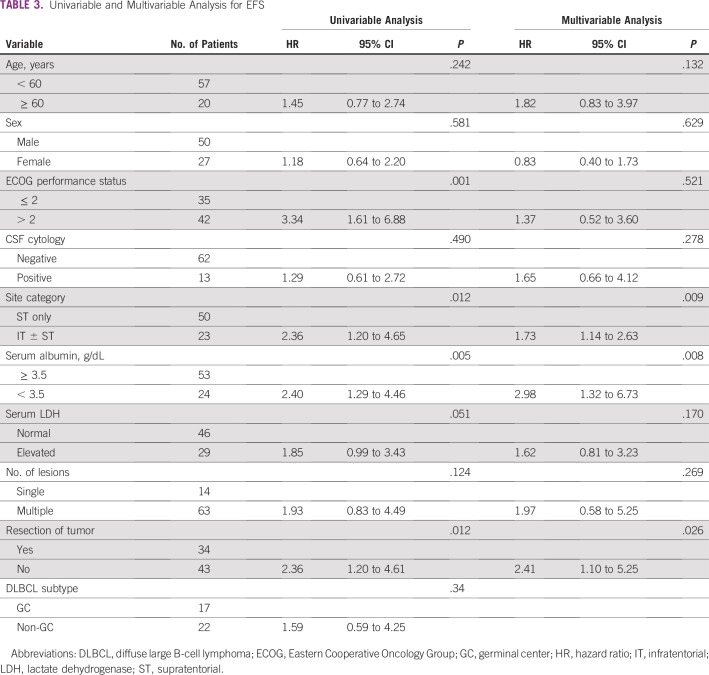
Univariable and Multivariable Analysis for EFS

**TABLE 4:**
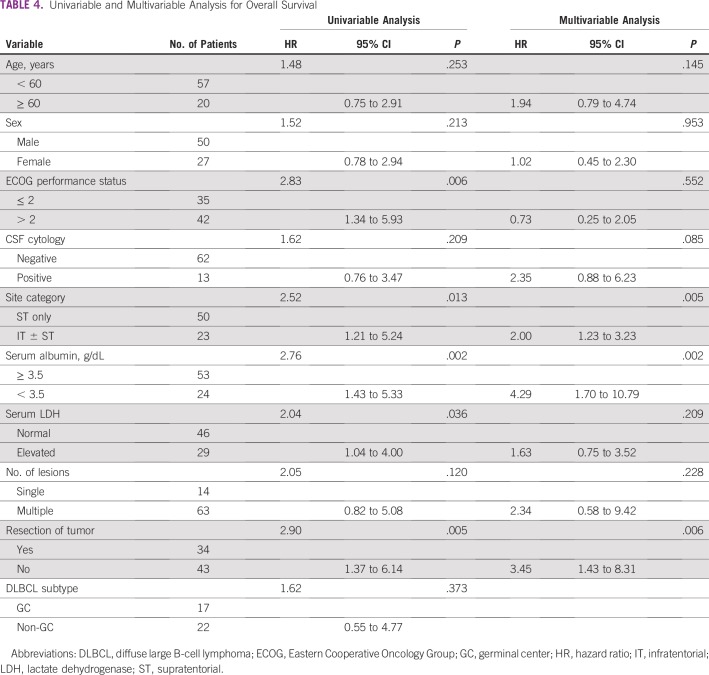
Univariable and Multivariable Analysis for Overall Survival

In 56 (72.7%) of 77 patients, the International Extra-Nodal Lymphoma Staging Group risk score could be calculated; patients in low-risk (n = 4), intermediate-risk (n = 32), and high-risk (n = 20) groups represented 7.1%, 57.1%, and 35.7% of the total, respectively (Table 1). The 2-year EFS for high-risk patients versus non-high-risk patients (n = 36) was 22% versus 65.7% (*P* = .006); the 2-year OS for high-risk versus non–high-risk patients (n = 36) was 32.7% versus 80.9% (*P* = .001).

We also did a subgroup analysis to evaluate the impact of rituximab (n = 27; 36.9%) when added to the MVP regimen (n = 73). Addition of rituximab resulted in higher ORR (88.9% *v* 73.9%; *P* = .12) and better achievement of CR (81.5% *v* 56.5%; *P* = .03). The median follow-up times in the MVP cohort with rituximab and in the MVP-only cohort were 20.7 months and 48.27 months, respectively. The 2-year EFS was better in the rituximab cohort (57.3% *v* 40.4%; *P* = .02), as was the 2-year OS (61.6% *v* 53.4%; *P* = .056).

There were 36 deaths in the whole cohort, of which 26 patients (72.2%) died as a result of either relapse or progression of PCNSL. Seven patients died as a result of unrelated or unknown causes, and three patients died as a result of chemotherapy-related toxicity. Among 17 patients who experienced disease relapse, 15 had local recurrences, and two had both local and systemic relapse. Most of the recurrences (12 of 17, or 70.6%) occurred within 2 years of diagnosis.

Treatment-related late neurotoxicity was observed in eight patients (10%) and was severe in two patients; in the rest, it was mild to moderate and was reversible in one case (normal pressure hydrocephalus, improved with ventriculoperitoneal shunt). In this cohort, late neurotoxicity was seen predominantly in the patients who received combined modality treatment (six of eight patients); five (62.5%) of the eight patients were age 60 years or older.

## DISCUSSION

This single-center retrospective study is, to our knowledge, the largest study to be reported from India, and it illustrates the difficulties and the challenges faced in the treatment of PCNSL in a developing country. The study is somewhat representative of the experiences across all parts of the country.^13,15,16,19-22^

In this study, 22 patients (22.2%) did not receive treatment because of poor PS (n = 14), refusal to take treatment (n = 5), or loss to follow-up (n = 3). The non-treatment rate is slightly more than the 8% to 15.3% rate reported in different studies from India.^19-21^ We noticed that the rate gradually decreased from 9% between 2001 and 2005 to 2% between 2016 and 2017; the reasons could be multiple (eg, timely diagnosis, better general condition, or PS).

In this study, we had only a single case of HIV-associated PCNSL (approximately 1%), which is similar to rates from many other reports from India—from 0% to 8.6%—including one autopsy study,^13-16,19-23^ and which is far less than the US incidence—from 26.2% (1980 to 2007) to 36.3% (1992 to 2011).^1^ The most plausible explanation is the earlier death of these patients as a result of opportunistic infections and tuberculosis.^16,23^

The median age among the immunocompetent patients in this study was 50 years; median ages reported by other centers in India, though, ranged from 42 to 59 years^14-16,19-22,24^ and reports from the West suggest that those patients are a decade older as well.^25,26^ This difference could be a result of the different age structure in India (only approximately 8% of the population is older than 60 years) or from the referral bias, but contribution of environmental or genetic factors cannot be excluded.

In this study, most of the occurrences were DLBCL (97.7%), and this was similar to other reports from India.^13-16,19-21^ By Han’s classification, non-germinal center type (56.4%) was more common in this study, but this was a small sample size (Table 2) (n = 39). A similar trend was also reported previously by us and others.^21,22,24,27^

In this study, the majority of the lesions were located in the supratentorial region, and the frontal lobe was the most common site—again similar to what is reported from other Indian studies^11,15,16,22^ and from Western literature.^25,26^ In this cohort, there were higher number of patients with high-burden disease compared with the Western literature, including multiple intracranial lesions (81.8% *v* 34%) and higher incidence of high intracranial tension features (49.4% *v* 33%) that reflected in the incidence of poor PS at presentation (PS of 4: 40.4% *v* 12%) in this study.^25,26^ The incidence of multiple intracranial lesions varied between 15% and 77% among different retrospective series from India^14,15,19-21^; in a prospective study from our center, multiple lesions were found in 75% of cases and reflected the influence of referral bias among different institutions across India.^17^

The rate of surgical resection in this study appears high (n = 40, or 45%) and mostly as a result of suspicion of glioma or other brain tumors (n = 36, 90%). In the rest, resection was performed because of the requirement of urgent decompression to improve the sensorium. A better EFS and OS was observed in the patients who underwent surgical resection in this study , as reported by a few other studies also,^28,29^ and the elimination of drug-resistant clones by surgical removal^10^ could be a theoretical cause. Better survival in patients who underwent surgical resection as compared with biopsyin the present study, is most likely due to a selection bias, and we refrain from deriving any conclusion from this finding and discourage resections in PCNSL, which is an infiltrative neoplasm.^30^

Toxicity from HDMTX and its relation to serum methotrexate concentrations is not as clear in PCNSL as it is in other malignancies, like osteosarcoma.^12^ At our center, we could not monitor methotrexate levels, because it is not available routinely. Thus, we had to rely entirely on adequate hydration, urine alkalinization, leucovorin, and monitoring of renal function and CBC. The mortality related to HDMTX in this study was 3.9%, which is comparable to another study (approximately 2.5% mortality) in which methotrexate levels were monitored prospectively in patients with PCNSL.^31^

In this study, the response to HDMTX-based chemotherapy with or without WBRT was 80.5% (CR, 67.5%), which is comparable to that of Western studies.^3,4^ There was an improvement in response rates—especially CR; EFS; and, to some extent, OS—in patients who received rituximab in addition to MVP. This improvement was similar to that seen in other reports, but longer follow-up is required for confirmation.^5,6^ At a median follow-up period of 34 months, the median EFS and OS were 20.4 months and 31.7 months, respectively. The outcome in this cohort was modest and can be improved upon. The impact of corticosteroids on delay of diagnosis and on the survival outcome in this cohort could not be formally addressed because of the lack of information about corticosteroid use. However, at least two thirds of patients had a history of prior use of corticosteroids, and these data will be prospectively analyzed. A better understanding of the molecular architecture of PCNSL may help integrate small molecules, especially ibrutinib, into the therapeutic armamentarium.^32^

Despite several inherent limitations related to the retrospective study design, this study demonstrated that most patients were young and immunocompetent but presented with a higher disease burden. The survival looks comparable to that of patients in the West but can still be improved upon with early diagnosis. However, it may be desirable to monitor the serum levels of methotrexate; at centers with no such facilities, HDMTX can still be administered safely but only with careful monitoring. At present, we need to put more effort into diagnosis of PCNSL in these patients early, which would certainly improve the outcome.

There have been numerous advances in the field of PCNSL during the past few decades, but notable barriers still remain with respect to identification and treatment of this rare, aggressive, yet curable brain tumor in the real world. More efforts are required to improve the care by formulating guidelines with respect to suspicion and diagnosis of this malignancy, development of cost-effective treatment protocols, improvement of supportive care, education and support of the community practitioners, and development of multidisciplinary care for these patients.

## Data Availability

The following represents disclosure information provided by authors of this manuscript. All relationships are considered compensated. Relationships are self-held unless noted. I = Immediate Family Member, Inst = My Institution. Relationships may not relate to the subject matter of this manuscript. For more information about ASCO's conflict of interest policy, please refer to www.asco.org/rwc or ascopubs.org/jco/site/ifc. **Research Funding:** CD Pharma India (Inst) No other potential conflicts of interest were reported.
